# Experimental Study of a 3D Printing Strategy for Polymer-Based Parts for Drone Equipment Using Bladeless Technology

**DOI:** 10.3390/polym16040533

**Published:** 2024-02-16

**Authors:** Florin Popișter, Horea Ștefan Goia, Paul Ciudin, Diana Dragomir

**Affiliations:** Department of Design Engineering and Robotics, Faculty of Industrial Engineering, Robotics and Production Management, Technical University of Cluj-Napoca, B-dul Muncii 103-105, 400641 Cluj-Napoca, Romania; florin.popister@muri.utcluj.ro (F.P.); ciudin.oc.paul@student.utcluj.ro (P.C.)

**Keywords:** 3D-printed parts, additive manufacturing, fused filament fabrication (FFF), complex shapes, bladeless technology

## Abstract

The present study focuses on an up-to-date topic regarding flying equipment identified within the category of drones that use, for propulsion and air movements, the power generated by electric motors. In this paper, researchers focus on implementing bladeless technology to calculate, develop, and construct flying equipment known in the literature as drones. The entire structure of the prototype, all the needed parts, is to be obtained using additive manufacturing technologies, which assumes practical realization using 3D-printing equipment. Nowadays, the 3D-printing process has been proven to be a reliable solution when it comes to manufacturing complex shape parts in quite a short time and with reduced costs. The practical study within the present research aims to obtain polymer-based, lightweight parts with complex shapes inside to be implemented in the propulsion of a drone. The complex surface geometry of the parts that this research used is influenced by the ventilation technology offered by the “Air Multiplier” technology. The entire structure of the final drone equipment, all the parts, is to be manufactured using fused filament fabrication (FFF). The main purpose of the fusion is to use the advantages offered by this technology in drones to obtain advantages such as augmented values of thrust, a more agreeable and muffled sound signature, or an increased level of safety.

## 1. Introduction

It is known that 3D printing, also known as additive manufacturing, is a transformative technology that creates 3D objects by adding materials layer by layer based on a digital model. This technology has found application across various industries due to its versatility, cost-effectiveness for small-scale production, and ability to create complex geometries. There are numerous technologies in the field of additive manufacturing or 3D printing. Fused filament fabrication (FFF) is one of the most common and widely used 3D-printing technologies, including in education, research, and prototyping. It is often referred to as fused deposition modeling (FDM), although the latter is a trademarked term owned by Stratasys, the company that originally developed this technology. FFF/FDM works by extruding thermoplastic filaments layer by layer to build up a three-dimensional object [1,2,3,4,5].

Currently, research on various types of poly lactic acid (PLA) materials has increased due to their potential use in industrial applications. Fiber-reinforced composites have a polymer matrix, where the fibers are typically made of synthetic materials, such as carbon or glass, due to their positive impact on the strength properties of 3D-printed parts. PLA, in combination with reinforcements or additives, can produce durable and functional parts suitable for different applications and developing prototypes [6,7,8,9,10].

**Figure 1:**
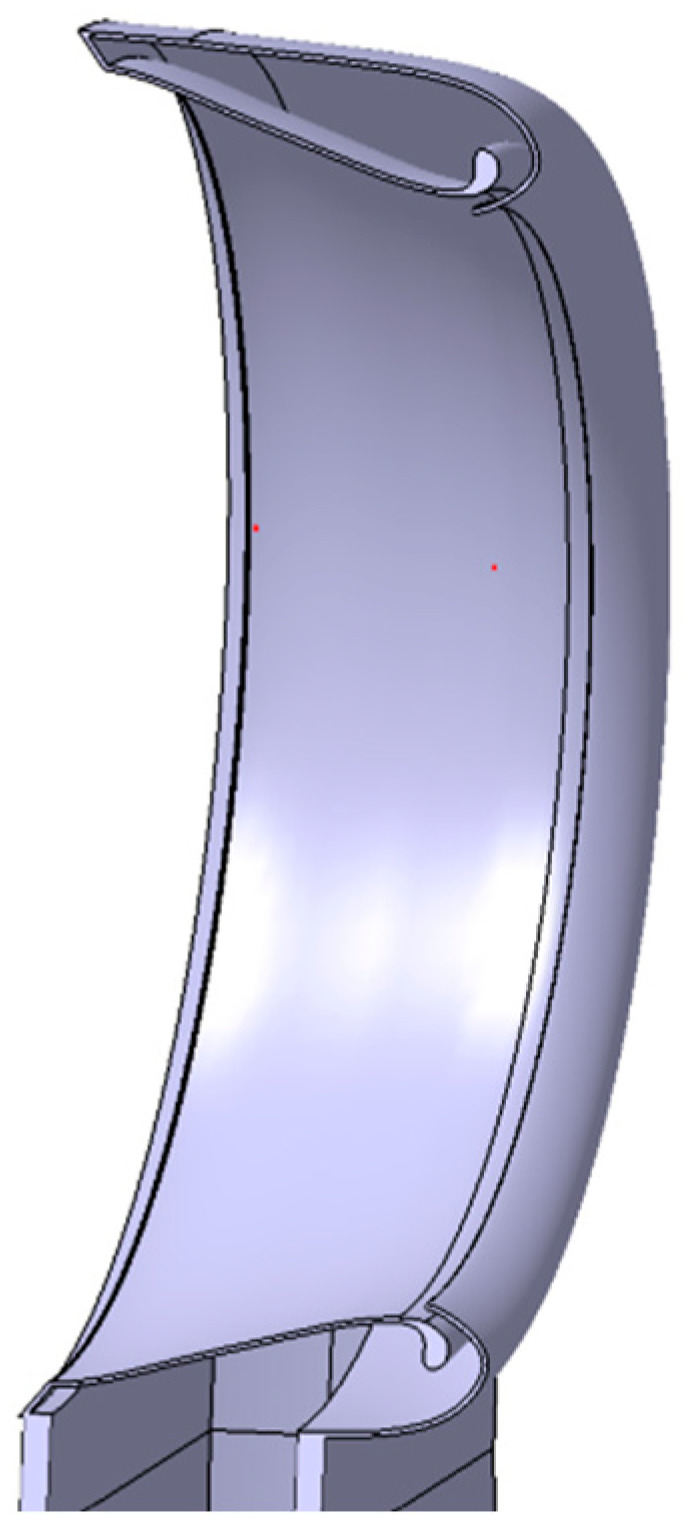
Section view, in accordance with the principle presented within the patent [11].

In the current research, the authors took into consideration the weights of the electronic equipment that are necessary for the 3D printed parts within the design process. Of the challenges that were faced within the current work, the biggest one was to obtain the main component, essential for this research, from a 3D CAD model—a part with complex geometry and width, presented in Figure 1, from the Dyson Air Multiplier technology (Dyson Technology Ltd., Singapore) further used for the liftoff a drone.

One of the defining features of [12] “Air Multiplier” technology is that the surfaces that the user may encounter do not have any moving parts that could facilitate an accident.

The amplified airflow achieved using the method outlined in this paper also leads to energy savings. By opting for airflow with flow and speed parameters within a range of values common to those of conventional fans, the amount of energy consumed will decrease as the fan operates in an economical mode. The fact that the model offers the possibility of low energy consumption is highlighted in [13] by drawing a parallel between the energy consumption of a fan based on Air Multiplier technology and a conventional fan.

**Figure 2:**
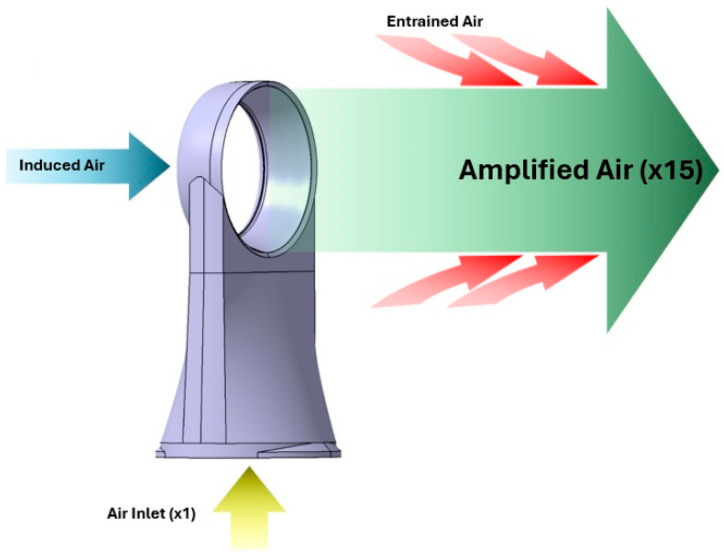
Airflow trajectory and phenomenon through the Dyson “Air Multiplier” functional geometry, as presented in [12].

Figure 2 shows, in an easy-to-understand way, the airflow path through the specific geometry and suggests the presence and action of the physical principles involved in its operation. Close to the rear of the unit, there is an element with dimensions that can have a significant effect on the speed, parameters, and operating efficiency of the unit. The element in question is the slot through which the air entrained inside the ring cavity flows, the parameters of interest affected by the slot width being the flow rate and the air flow velocity. Immediately after exiting the slot, the air follows a trajectory adjacent to the inner surface of the ring because of the effect observed by the aerodynamics specialist Henri Coandă, which leads to a low-pressure zone, coaxial with the center of the ring.

This area of low pressure creates an induction phenomenon, which is manifested by airflow entering the ring of the structure through the rear plane. The inertia of this air stream causes it to continue its path even after exiting through the front plane, forming a cumulative flow with the air expelled through the slot, thus generating the first stage of flow multiplication. The second stage of flow intensification is due to the phenomenon of air entrainment in the vicinity of the exit jet. The veracity of this phenomenon was also studied and analyzed in [14], “The vortex ring state of a rotor and its comparison with the collapse of an annular jet in counterflow”, published in “Physics of Fluids” in early 2023.

## 2. Theoretical Aspects

**Figure 3:**
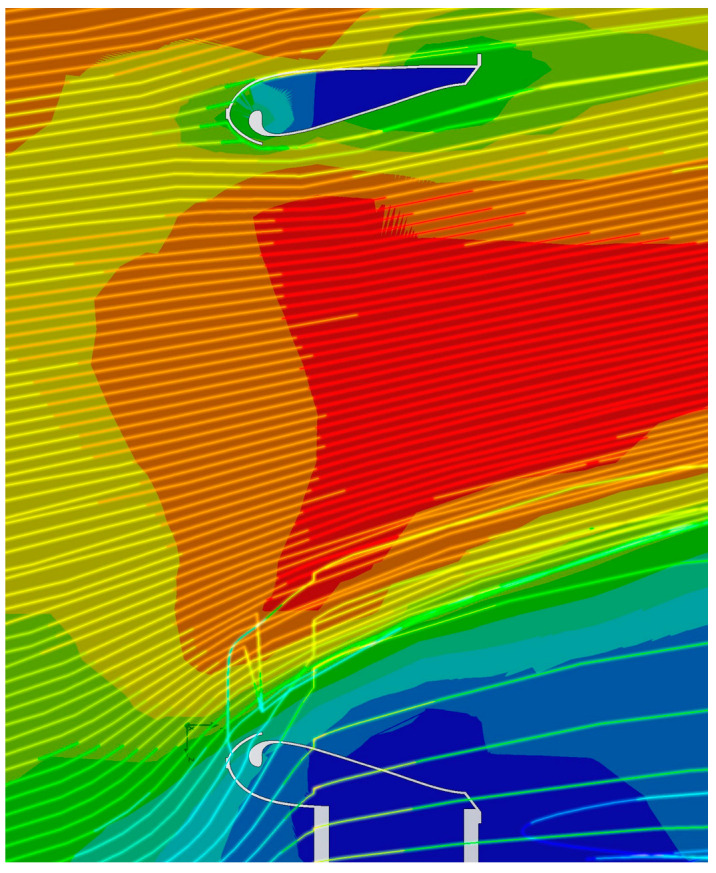
Distribution of velocity, marked with red, presenting higher values within the fan enclosure according to [15].

In 2014, a group of researchers from Tehran’s Sharif University of Technology conducted a study [15] around the concept of a propeller-less fan. The topic was approached using both theoretical and computational methods, as well as practical experiments. Gravitating around the operation and efficiency of the phenomenon, concurrently with the analysis of the noise level produced by its operation, the researchers pursued the feasibility of using a propeller-less fan in the 1000 Hz industrial environment, which justifies the efforts of the development team of the company “Dyson Technology Ltd. Singapore”, by adding the “Helmholtz” cavity to decrease the noise level of the “Dyson” fans. The results of the study show that of the 100% of air generated, 8.5% is the amount directly introduced by the propeller drive, 53% is due to the phenomenon of induction, and the remaining 38.5% comes from the environment through adherence to the air mass due to the turbulent currents generated downstream of the fan. The value of the total multiplication coefficient obtained by the researchers was 11.5 for the numerical method and 13.5 for the experimental method, respectively. Figure 3 depicts the air trajectories in the case of the fan body, as presented in the mentioned paper.

Other external entities have also approached the concept of “Air Multiplier” technology, leaning toward a more practical approach, such as conceptual models, prototypes, or even mass production of their products. Among these, we can mention the case of [16] “Jetoptera”, which is currently at an advanced stage in the process of testing and improving a flying vehicle with VTOL capabilities intended for mass use. A more theoretical approach has been taken by a team of researchers from the “Hindusthan College of Engineering and Technology”. In their study [17], “An Overview of Bladeless Drone”, the team presents a quadcopter concept that uses “Air Multiplier” technology to enhance the capabilities of conventional UAVs. Another attempt to exploit the advantages offered by the ventilation technology mentioned above was made by a team of researchers from Guangdong University of Science and Technology. After designing a quadcopter drone with specific thrusters based on the “Air Multiplier” principle, they concluded, in the context of a [18] scientific article, on the high potential of this propulsion method for UAVs. Similar conclusions to those presented in [18] are reached in [19], “Improving Safety: Design and Development of a Bladeless Thruster for Autonomous Multicopters”, which, like the present study, aims to establish a solution for achieving rotational motion around the vertical axis, YAW. Opting for a quadcopter-type motor structure, the solution chosen by the authors of the study was to implement mechanisms for rotating the propulsion structures around their longitudinal axes located in the horizontal plane, controlled by means of PID control structures.

Supplementarily, within the current work, the authors elaborated and carried out calculations both for the mechanical part and for the aspects that are the object of the actual construction of the prototype, i.e., calculations related to the motors as well as finite element analysis calculations. All the above-mentioned calculations have been developed in dedicated applications such as MathCAD and Catia V5.

### 2.1. Calculus of the Motors

In the design process of a model drone, the correct sizing of the electric motors is crucial, as the designer must consider a variety of factors and parameters that depend on the capabilities of the motors. These include the ability of the motors to generate sufficient force for the proposed flight capabilities and to withstand long flight sessions while keeping the weight of the assembly as low as possible and to keep costs down, both in terms of production and operating costs

- Gravitational force.

The initial value to be calculated is that of the weight force of the assembly using Formula (1):

(1) F_G=m×g

where F_G is the gravitational force acting on the prototype assembly, m is its mass, and g is the gravitational acceleration constant (9.80 $m/s^{2}$).

Given the prototype nature of the assembly in question, its mass is measured using an electronic scale: m = 954 g. The value of the gravitational force is thus

(2) F_G=9.73 N

- Drag force.

In the field of fluid mechanics, the drag force is the force that an object encounters when moving through a fluid, which is given by Formula (3), as offered in [20], a course by “Lumen Learning”.

(3) $$F\_drag=1/2\times \rho \times v^{2}\times C\_d\times A$$

where F_drag is the drag force against the air; *ρ* is the air density, with a value of 1.225 kg/m^3^; v is the lift velocity imposed at take-off, found in the usual case of drones at 2 m/s; C_d is the drag coefficient of the air; and A is the contact surface with air.

Given the shape of the main body of the drone observed from above, according to the drag coefficients as a function of shape from [21]—the publication by Dr. Sighard F. Hoerner, “Fluid-Dynamic Drag”—for the value of the parameter C_d, a value of 0.6 was chosen, which is less aerodynamic than that of a streamlined body (0.07). Due to the secondary components of the assembly, such as the lateral thrusters or the support structures, the chosen value falls within the range of values of the coefficients of drag that are specific to spheres and cylinders.

The value of the contact surface area was measured on the 3D model of the concept using the CATIA V5 solution. This resulted in the following value: A= 0.394 m^2^.

This gives the value of the drag force through the air as

(4) F_drag=0.57 N

- Total force required.

The total force required to be generated by the drone’s engines to achieve lift is calculated using Formula (5):

(5) F_(total required)=F_G + F_drag

(6) F_(total required)=10.3 N

- Motor rotation speed (at the shaft).

To determine this value, it is necessary to know some specific parameters of brushless electric motors available on the market. In this regard, the MT2204 motor model is proposed, which, as part of an electronic circuit powered by a battery supplying 7.4 or 11.1 volts, has the speed constant K_v with a value of 2300 RPM/volt.

This value is essential for determining the shaft speed of the motor, and Formula (7), mentioned in [22], an “Astro flight” article, is adapted to include its shaft parameters.

(7) K_(v(shaft))=(K_v·(2π·r)/60)/(1 V)

where K_(v(shaft)) is the shaft speed of the motor and *r* is the radius of the shaft on which the propeller is mounted, which is taken from [23], the documentation provided by the manufacturer, having a value of 1.5 mm, equivalent to 0.0015 m.

After replacement, the value of the motor shaft speed is as follows:

(8) K_(v(shaft))=0.361 (m/s)/V

Having obtained this value, it can be stated that the motor can develop speeds of 2.67 m/s when supplied with a voltage of 7.4 volts, and 4.01 m/s when supplied with a voltage of 11.1 volts, respectively, both architectures being feasible for the motor design. The calculations continue using the value of 4.01 m/s, the less favorable variant.

To obtain the speed value in terms of rotational speed, we divide the previous speed value by the radius of the shaft, as shown in Formula (9).

(9) K_(v((rad/s)/V))=(K_(v(m/s))/r)/V

(10) K_(v((rad/s)/V))=240.7 (rad/s)/V

Considering the voltage value of 7.4 volts, the rotation speed of the shaft is calculated using the following formula:

(11) K_(v(RPM))=K_v((rad/s)/V)·V·60

(12) K_(v(RPM))=106,870.8 RPM

### 2.2. Presentation of the 3D Model

**Figure 4:**
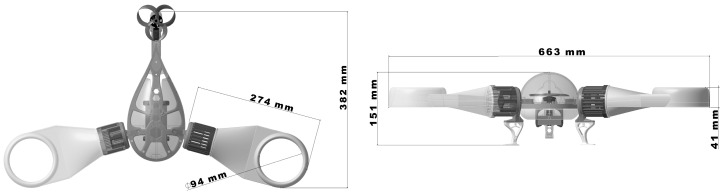
The concept’s 3D assembly model.

In addition, the three-dimensional assembly, shown in Figure 4, was developed with aerodynamic aspects in mind, as distinguished by the drop shape of the main body. Finally, given the large number of elements, the assembly methods required are diverse, the model being fitted with elements contributing to the rigidity of the assembly.

**Figure 5:**
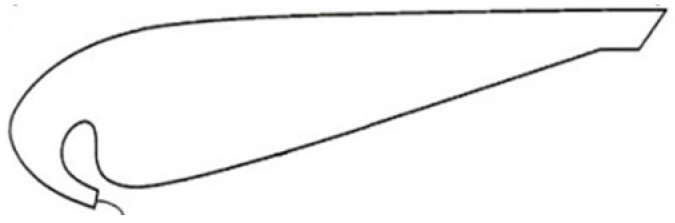
Cross-section of the “Air Multiplier” technology, extracted from the “Experimental and Numerical Investigation of a 60 cm Diameter Bladeless Fan” study [15].

The modeling process of the assembly started with the geometric elements related to the technology developed by “Dyson Technology Ltd., Singapore”. The central element is one that incorporates both the ‘Coandă’ surface and the inner surface of the fan. To achieve greater fidelity in the original model, the sketch module was used to design it. The first iteration of this geometry is shown in Figure 5. The resulting geometry was produced at a scale of 1 in 10 compared to the prototypes made in [15], the previously mentioned study, to adapt it to the dimensional requirements of a drone. The profile sizing is also good according to the data presented in the scientific paper [24] “Development of Bladeless Thruster for a UAV Application”, which focuses on the study of the parameters of a structure inspired by the “Air Multiplier” technology by means of theoretic analysis, CFD computer simulation, and prototyping methods.

Found in all drone structures, propellers are the interface between the rotational movements of the electric motors and the air currents being handled. Although the genuine “Air Multiplier” model uses impellers, which guide the air currents in lateral directions to minimize collisions between the air and the inner walls of the geometry, the proposed model has a larger air access path inside the circular shell, which allows for the replacement of the originally designed impellers with propellers.

**Figure 6:**
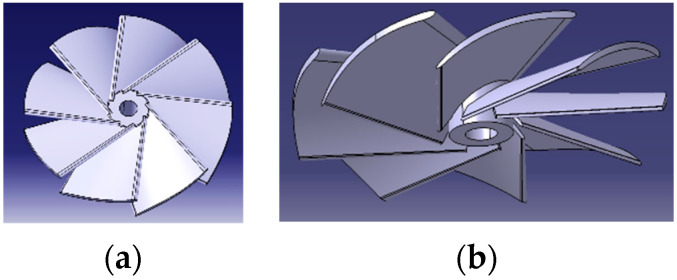
The proposed model of propeller: (**a**) top view; (**b**) isometric view.

The process of selecting the propeller model for driving the air currents inside the front thrusters began with a study of their functional parameters, followed by a study of the variants available on the Internet. After several attempts, an original propeller model was designed, exploiting the knowledge gained to obtain a propeller that met the specific requirements of the application, as shown in Figure 6.

The analysis of Figure 6 allows us to observe the vertical plane profiles of the pallets, as well as their number. The eight elements have a considerable width in relation to this plane and are partially interleaved. This has been utilized to avoid vibrations and to increase the upper limit of the possible speed range, the air masses being more evenly distributed.

The difference between the thicknesses of the two blade edges contributes to the occurrence of the Coandă effect and the enforcement of Bernoulli’s law, making it plausible for zones characterized by low pressure to form on the upper surfaces of the blades.

There is a close connection between the propulsion principle chosen for the proposed concept and fluid mechanics, both having a distinct way of exploiting the geometry in order to manipulate the trajectory of the air currents and therefore their parameters. In order to provide a more efficient way of visualizing the concept and an easier understanding of it, computer-assisted simulations of the geometry and behavior of the air volumes in its interior were performed.

The simulations were carried out using the Flow Simulation module of SolidWorks2022, considering the properties of the materials used in the prototyping process, ABS and PLA polymers, to ensure the accuracy of the design. Further boundary and operating conditions, as well as results, are presented in Table 1 in the case of currents adjacent to the outside annular structure of the thrusters.

**Figure 7:**
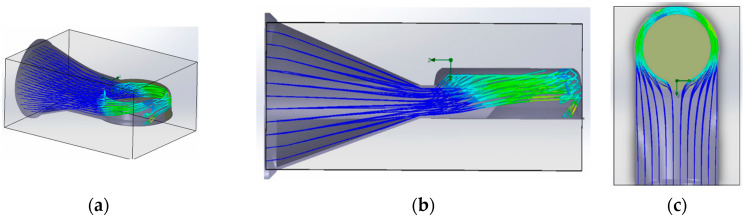
Trajectories and velocity values of the air through the geometry: (**a**) isometric view; (**b**) top view; (**c**) side view.

In addition to the possibility of visualizing the behavior of the air currents inside the geometry, Figure 7 makes it possible to observe the amplification of their velocity from the point of incidence with the inner volume of the ring, confirming at the same time the theory relating to this section of the geometry of the “Air Multiplier” technology, as the volume of air is pushed and extruded through the marginal slot of the “Coandă” surface. The same figure also makes it possible to observe the behavior of the air currents in the area opposite their source, inside the ring.

The simulation of the flow behavior of the air jets surrounding the annular structure of the propulsion unit is of interest to confirm the veracity of the induction and entrainment phenomena, which are due to the amplifying effect of the dynamic parameters of the entrained fluid.

**Figure 8:**
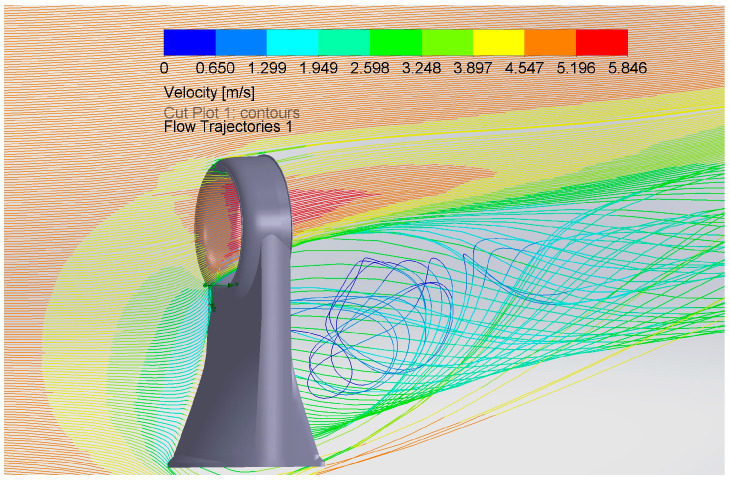
Trajectories and velocity values of air currents adjacent to the annular structure of the thrusters.

An analysis of Figure 8 confirms the presence of an area characterized by a pronounced velocity index located in the central area of the volume surrounded by the circular “Coandă” surface, which attests to the manufacturer’s claims.

**Figure 9:**
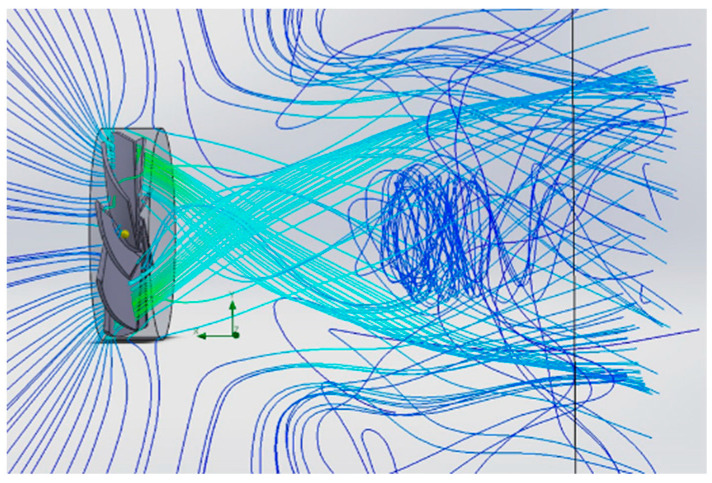
Velocity values in relation to the flow trajectories generated using the designed propeller.

The propellers used in the front thrusters, the model of which was designed in this work, are the main mechanical elements. In order to dimension them and in accordance with the specific “Air Multiplier” technology, which is the basis of the thrusters’ operation, an “air flow” simulation was carried out using the same solution offered in SolidWorks2022. The propeller model in question was loaded, and the geometry of the volume in which it is turned was determined. After using specific computational methods and considering the conclusions of the article [25] “Study of Mesh Quality Improvement for CFD Analysis of an Airfoil”, the values shown in Figure 9 were obtained, considering the input value of the rotational speed of 942.5 radians per second. The green to turquoise shade of colors in Figure 9 represent the differences in velocity of the air flow that is generated by a propeller in a decreasing trend. 

The weight of one cubic meter of air, according to [26], at a normal temperature of 20 °C and atmospheric pressure of 101.325 kPa, is 1.2 kg. We can thus calculate the force generated by the air currents using Formula (13):

(13) F=m×v

where m is the mass of one cubic meter of air and v is the velocity of the air currents, having the value of 7 m/s, as a result of the simulation.

(14) F=8.4 kg×m/s

The next step is to transform the force value, from kg m/s, into N×s, thus deriving the force value of

(15) F=8.4 N×s

Considering the multiplication effect proclaimed by the manufacturer of the “Air Multiplier” technology, we can thus estimate the final thrust value as being 126 N×s.

## 3. Practical Development of the Drone

The present study proposes to analyze, optimize, and use appropriate techniques, methods, and parameters related to fused filament fabrication that would ultimately be applied in the prototyping process of a device that exploits the synergy between the ‘Air Multiplier’ ventilation technique developed by Dyson Technology Ltd. Singapore and the concept of drone equipment. The theoretical study of the concepts allowed the formulation of several requirements to be met by the proposed model. The following requirements are identified for the fused filament manufacturing process and are imposed by the initial concept developed by the authors:

1. The structure must exhibit a high level of rigidity.
2. The assembly must have a low mass.
3. Surface quality must be adequate, particularly on surfaces in contact with air currents.
4. Components must be capable of withstanding specific loads.
5. Prototyping should prevent issues from arising at contact surfaces with components or fasteners made from different materials.
6. Efficient and easy assembly must be facilitated by the surfaces on which the attachment is carried out.

In addition to the requirements related to the fused filament fabrication method, the developed drone model must have features common to all aircraft in this class. The following objectives are thus distinguished:

1. The propulsion system of the assembly must provide superior thrust force values compared to those provided by the established method used by drones currently available on the market.
2. The motion model must provide dynamic behavior capabilities at least equivalent to those of current multi-rotor type drones.
3. The sound signature of the concept should exploit the quietness of the “Air Multiplier” technology, offering the alternative of a flying machine with a reduced noise level.
4. The level of safety required must be high, given that the blades are embedded in the propeller body.
5. The assembly must offer a low energy consumption for an equivalent value of propulsion force compared to usual drones.
6. The assembly must offer good maneuverability.

The synergetic model proposed in this paper is as follows: two air-amplifying geometric structures in the front area, acting in consensus, together with a motor on the axis on which a propeller is mounted, found in the rear area. As in the consecrated solution, all the above are obtained with the facilitated advantages offered by the additive manufacturing process—3D printing.

The motor model of the tricopter drones offers the possibility of integrating the functional geometry of the airflow amplification, thanks to the unique element that gives the “YAW” mode of movement. This is the servomotor mechanism whose role is to tilt the rear motor according to the desired direction of rotation around the vertical axis. 

In keeping with the tricopter drone design, the propulsion structures, like Dyson fans, are in the front area, forming a 140-degree angle between them. Starting from the area close to the drone’s body, the geometric structure of the front thrusters can be divided into three zones. The first of these has the structural role of establishing contact between the body of the UAV and the rest of the multiplying geometry, inside which, embedded in a slotted cylinder, is the support for the electric motor. Through these slots, a propeller designed to maximize the volume of air entrained, mounted on the motor shaft, draws air from the external environment and entrains it to the next area. The second zone acts as an adaptor between the peripheral sections, adapting the shape described by the air flow from the circular section of the motor enclosure to the seemingly rectangular one required to properly pressurize section three. The third section is the geometry required for the induction and entrainment phenomena.

### The Prototyping and Assembly of the Model

Regarding the additive prototyping process, it is worth noting that the difficulty of accurately controlling the dimensions of the molten filament when it is deposited on the surface of the previous layer means that it is possible to obtain significantly larger dimensions, which is a problem for functional components or parts where dimensional tolerances are especially important.

Given the stage of the study and the prototype nature of the practical work, for the FFF process, ABS (Acrylonitrile Butadiene Styrene) and PLA (Poly Lactic Acid) polymers have been chosen as base materials. The current prototype is in its early stages and aims to demonstrate the applicability of Air Multiplying technology in drone propulsion systems, as well as the advantages of FFF processes. Further improvements and optimizations are reserved for future stages of development, when more specialized types of polymers will be used, offering superior properties to the parts, such as better tensile strength, lower mass, or improved malleability. The ability to prototype parts presenting complex shapes in a short period of time while providing good properties for the structure is one of the main advantages offered by the FFF techniques over classic means of production. The choice of polymers used in FFF also offers advantages such as low cost of acquisition, wide market availability, and ease of use.

Relevant parameters of the manufactured composing parts of the prototype, as specific to the chosen polymers, are indexed in Table 2 in accordance with the results of specialized tests [27].

**Figure 10:**
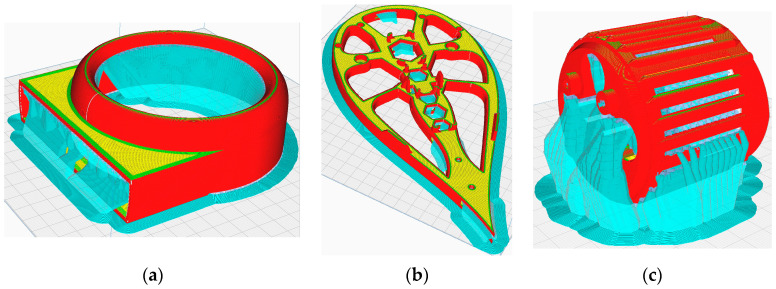
(**a**) Outside Air Multiplier Cylinder; (**b**) Base Part; (**c**) Motor Cage.

FFF permits the manipulation of certain structural parameters of the resulting parts using dedicated slicing software, which, in accordance with the specifications of a 3D printing machine, can generate the source code for prototyping a part. In the process of developing the prototype, the slicing software that was utilized was “UltiMakerCura 5.2.1”. Figure 10 presents the simulated process of prototyping various parts of the assembly offered by the mentioned software to facilitate the visualization and validation of the code.

The parameters that can be adjusted using the slicing software affect the final product in various ways, according to different needs. Table 3 presents various parameters and their corresponding values assigned for three relevant parts of the assembly. The Layer Height, which has a relatively low value, affects the surface quality, which is an important factor in this case. The values of Wall Line count and Infill Density were manipulated for the parts to have reduced values of mass, yet with good structural integrity and strength. Printing temperatures are closely related to the type of filament. For the used polymers, the best results were obtained for the given values of printing temperature and Build Plate Temperature. For complex shapes, the tree type of Support Structure has proven to be useful. Additionally, to print the thin walls of the Air Multiplier technology, the Thin Walls option must be activated.

The strategy for prototyping the drone components aimed to create lightweight yet durable structures that meet the specific requirements of a drone flight apparatus. To realize this, the printing parameters are modified by reducing the infill and increasing the wall layer count. An additional benefit is the superior shock-absorption capability, as these structures allow the bodies to have some flexibility in the face of possible shocks.

**Figure 11:**
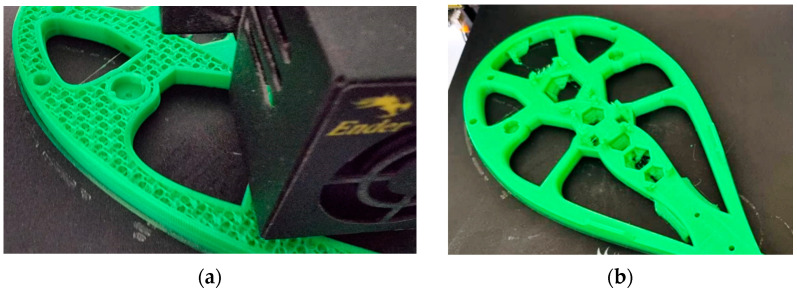
(**a**). Intermediate layer of the base part. (**b**) Prototype’s finished base part.

For the first of these, Figure 11 shows the printing process of the base part, both during the process where the internal structure of this part can be observed and after the process has been completed.

According to [28], one of the main disadvantages of FFF/FDM prototyping techniques is their low dimensional accuracy. This issue affects the structure in various ways and at different times.

One positive aspect is the ease of mounting the electronic modules used to operate and control the drone’s electronic structure; in fact, a low level of dimensional tolerance allows the electronic modules to be mounted more efficiently in relation to the designed support elements, without the need to design more complex mounting structures.

For future prototype iterations, the mass of the base part could be further reduced, while the PCBs could be mounted more seamlessly within the structure of the base. More flexible electronic boards [29] would be able to be mounted more efficiently, having more similar properties to the material of the base than the classical PCB materials, which necessitate more complex mounting elements or methods because of the difference in rigidity. Furthermore, this type of piezoelectric polymer could better resist shocks, potentially reducing the maintenance costs of drone devices. Additionally, converting vibrations into electrical energy could even help increase the device’s battery life. Efforts to increase the flight time of such devices and to miniaturize the electronic components and the overall mass of the assembly could also be helped by using the latest technologies in the field of batteries. Miniaturized energy storage devices, and even 3D-printed ones [30], offer the potential for a breakthrough in the field of flying devices such as the presented prototype. Both studies present the possibility of integrating elements of the electronic structures directly into the body of the base part by means of FFF.

The potential advantage of FFF prototyping in obtaining a suitable volume for self-tapping holes can also be applied to the screw assembly. However, it is crucial to consider the orientation of the layers in the structure that contains the screw mounting holes. The layers must be arranged in a plane that competes with the screw-turning axis to enhance resistance to shear phenomena at the thread level during tightening.

**Figure 12:**
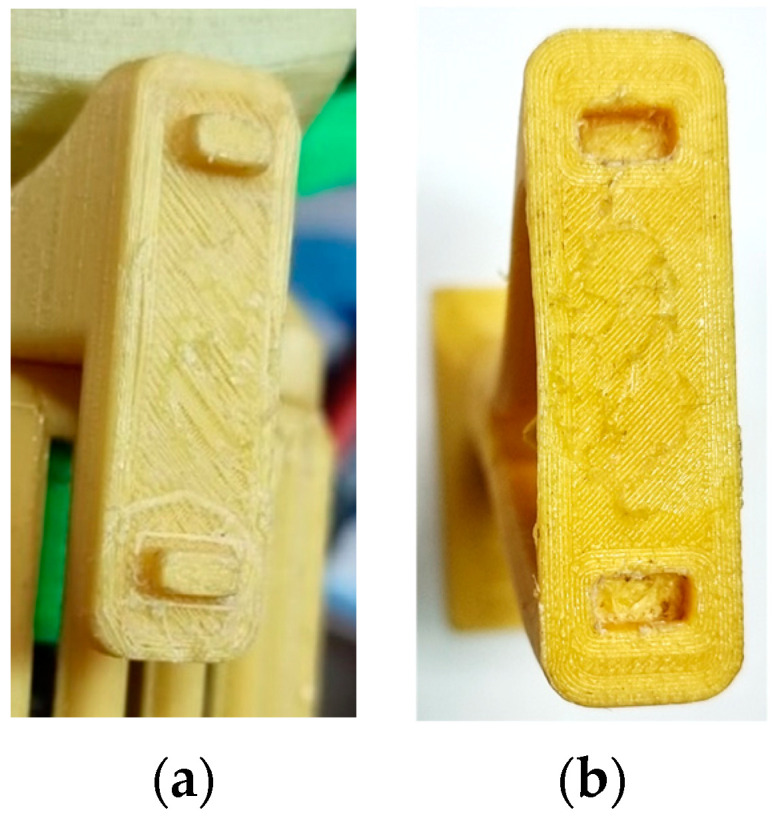
Assembly interface of the front support elements. (**a**) Assembly side, (**b**) support element side.

During the assembly process of the prototype, a major issue was encountered at the mounting interface of the drone’s front support elements in relation to the main assembly structure. The tolerance values were critical in achieving a correct assembly (see Figure 12a, the assembly side, and Figure 12b, the support element side, for a visual representation of the interface).

**Figure 13:**
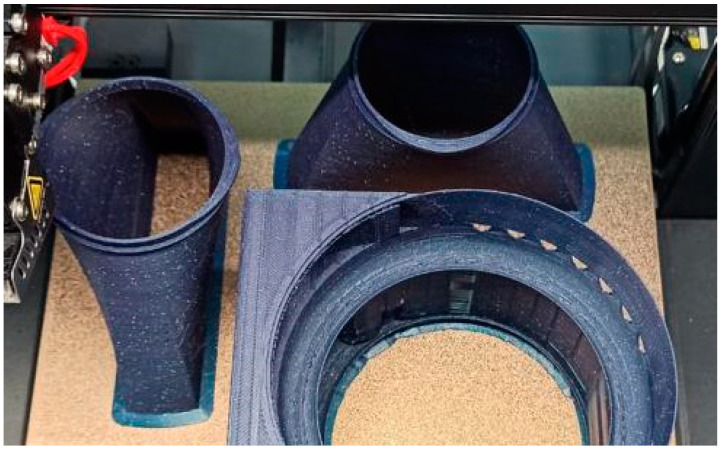
Air-guiding funnels at the end of the printing process.

**Figure 14:**
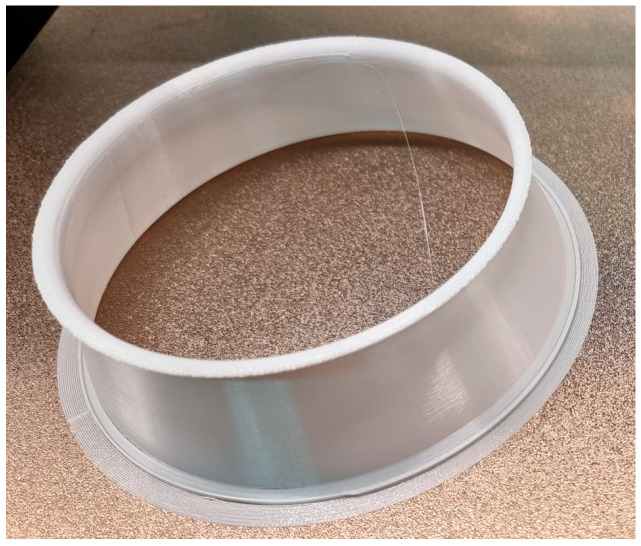
The “Coandă Surface element” at the end of the printing process.

The 3D printing of the geometry specific to the “Dyson” technology was a challenge due to the complex shapes and the inner channel for the passage of air; therefore, the structure was divided into three parts: the funnel that guides the air from the motor area to the rest of the geometry, the outer shell of the circular section, and the circular wing-shaped structure, also called the “Coandă” surface, respectively. Figure 13 shows two of the component models of the geometry associated with the Air Multiplier technology, namely, the funnels in the vicinity of the motors at the end of the 3D-printing process. The last of these, the “Coandă” surface element, captured at the end point of the printing process, is shown in Figure 14.

The layers of these elements were oriented based on several criteria. The chosen orientation, visible in Figure 13 and Figure 14, aims toward an airtight assembly and maintains the size of the air outlet slot around the entire circumference of the slot. However, this orientation also has some disadvantages.

From a structural perspective, using this layer orientation to create thin walls poses risks to structural strength. Figure 13 also shows a prototyping defect in an early prototype, which may be due to a combination of factors such as thin walls, layer orientation, or errors in machine code generation.

Although air multiplication technology is known for its low noise level, imperfections in the interior surfaces adjacent to the airflow can negate this benefit. The surface roughness of the FFF technique, combined with the chosen layer orientation, does not significantly decrease the noise emitted during the prototype’s operation, as expected from this technology. Instead, it has a trade-off character between the two opposite effects.

Surface quality is a crucial parameter to consider when employing Air Multiplier technology in a drone’s propulsion system, as it is necessary to minimize friction between the airflow and the internal walls of the geometric structure. To obtain smoother surfaces in such cases, coatings or chemical treatments can be applied. Maintaining a low mass of the assembly, along with the complex geometry imposed by the Air Multiplier technology, by applying a coating is a less-than-ideal solution. Studies, such as [31], have shown that chemical treatments can reduce the elasticity and strength of 3D-printed parts. Considering these aspects, this study has prioritized the structural integrity of the assembly; therefore, surface treatments have not been applied.

**Figure 15:**
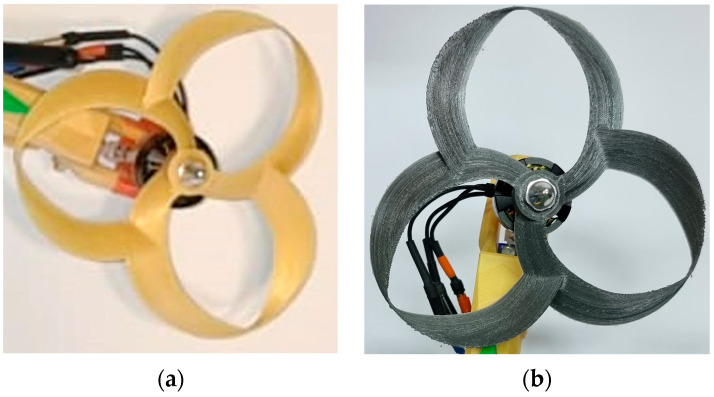
Toroidal propellers: (**a**) first instance (ABS), (**b**) second instance (polycarbonate).

Given the difference between the propulsion principles chosen for the front and rear motor structures, the propeller model mounted on the rear electric motor shaft was developed by MIT University researcher Thomas Sebastian [32], as shown in Figure 15. The choice of this solution is justified by the superior propulsive capabilities and safety level, together with the reduced emitted noise, that the toroidal propeller model presents, according to [33].

The toroidal propeller’s initial prototype was printed using a generic ABS material. During testing, it failed due to the stress it was subjected to. Using the results presented in [34], polycarbonate filament was used to fabricate the element. This was due to the inconvenience of modifying parameters such as layer orientation while maintaining a low mass. The use of polycarbonate increased the tensile stress values from 28.75 MPa to 49.08 MPa, values provided by the authors of the article. The comparison of the two instances can be seen in Figure 15.

The assembly of all these elements was carried out using the designed fixing elements and the techniques provided by the computer-aided design solution CATIA V5. Therefore, the rear leg, together with the support element of the rear motor steering mechanism, was fixed to the base by means of a nut and bolt. The shaft of the moving element of the mechanism was mounted in the bore designed in the support element, and the servomotor was fixed using two nuts and bolts.

The propellers used in the front thruster structures were prototyped, as shown in Figure 6b. A specific feature in terms of the FFF technique was the orientation of the layers in a plane perpendicular to the axis of rotation of the element to increase the flexibility of the lower part of the blades, which is deliberately not in contact with the mounting hub of the structure. These two aspects increase the adaptability and strength of the geometry when operating at high rotational speeds.

**Figure 16:**
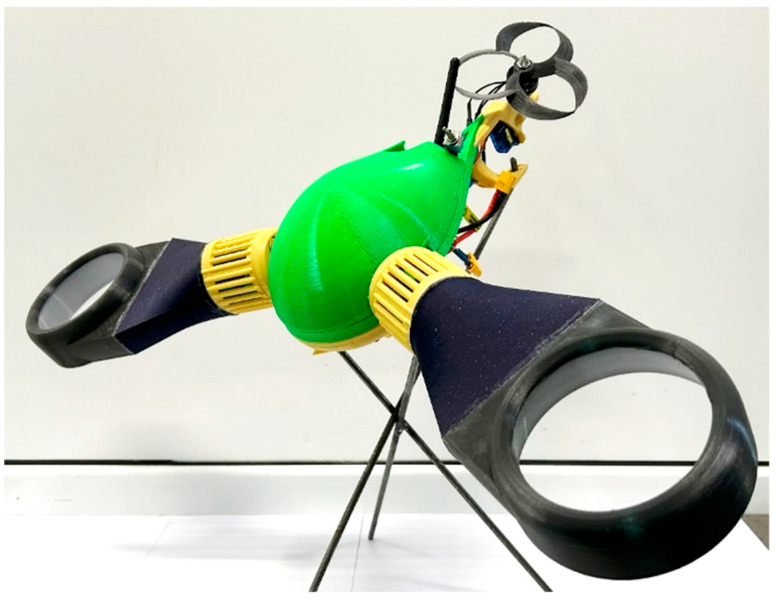
The 3D-printed prototype.

The front motors are mounted in the manner specified by the manufacturer, and the propellers are fixed to their shafts using the threaded element provided for this purpose; the final assembly of the prototype is shown in Figure 16.

**Figure 17:**
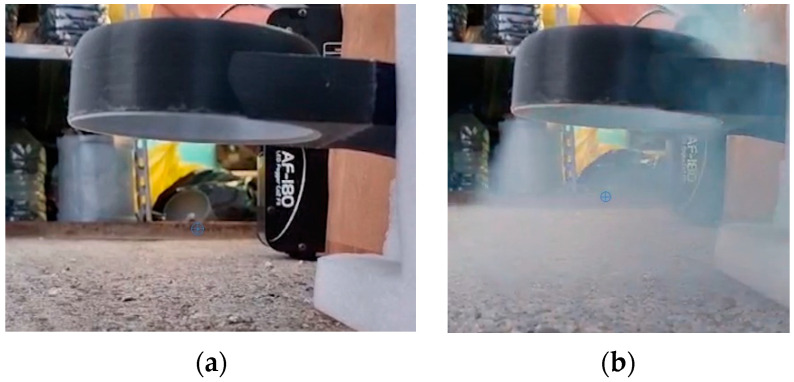
Testing the airflow using a smoke machine in (**a**) motors off case, meant for comparison, and (**b**) the case in which smoke is entrained through the structure of the fan.

To validate the thin, complex shape of 3D-printed parts, several tests were conducted using the smoke machine seen in Figure 17 in order to establish if the proposed idea and final parts corresponded to the initial form of the concept. The result of the test revealed that the printed parts resemble the theory and practical calculations developed by the authors.

The assembled prototype comprises protective elements for the electronic structure. These include an upper cover in the shape of a half water droplet that shields the electronic boards and an air deflection and battery protection element located in the lower front area between the two front propulsion structures. Currently, both elements are made of an ABS material with relatively thin walls, which poses a significant risk of damage. A potential improvement for these components can be fabricated using polymers with lower stiffness, such as nylon, TPU, or Soft PLA. This would increase their shock-absorbing capabilities without compromising their efficient air deflection or protection capacity.

Another improvement direction is for a larger number of the assembly’s component parts to be remanufactured using different materials in order to decrease the overall mass of the aircraft but without decreasing its strength parameters. Despite the possible difficulties in the prototyping process, viable materials for this process include polycarbonate (PC), Polyaryletherketone (PAEK), Poly-aryletherketone (PEI), or composite filaments, such as carbon fiber-reinforced filaments.

## 4. Conclusions

The current state of the proposed topic of this study can be concluded from the results obtained so far, both in terms of theoretical conclusions and results presented in the form of the prototype. These are relevant, despite the relatively early stage of the product development process, due to its scale and complexity in relation to the finality of releasing on the market a product based on the proposed concept. This perspective requires a large additional volume of experimental activities, variations of the prototype, and the allocation of a significant quantity of resources. In the initial phase, it will be used as an educational element that connects knowledge together with expertise from the partners through dedicated industrial competitive development [35].

The prototype developed as part of the practical part of the study proves the possibility of implementing the proposed concept of movement through a structure with low weight in relation to the materials and prototyping processes used. Also, the design uses some characteristics of the canonical model of drones, such as the angle between the main engines, while at the same time presenting aspects relating to the aerodynamics of the flight assemblies through the shape of the structural elements. In addition, the structure’s functional capability can serve as proof of the overall validity of the design and prototyping aspects.

Another conclusion can be drawn from the adjacent aerodynamic concepts. The prototype exploits the advantages of fitting the propellers inside the tubular sections of the thrusters without the method of prototyping encountering significant impediments. The propellers used for the front thrusters have been designed to maximize the entrained air volume in accordance with the studied concepts and the aspects related to the FFF prototipation. The efficiency in terms of safety, thrust, and noise signature of the toroidal thrusters has been tested using such a propeller on the rear engine shaft.

## Figures and Tables

**Table 1:** Boundary conditions and results of the airflow simulation.

Basic Mesh Dimensions	Nx = 27, Ny = 52, Nz = 26
Pressure [Pa]	101,305.44 to 101,345.97
Velocity [m/s]	0 to 5.846
Gravity	Yes
Temperature [K]	293.19 to 293.21
Density of Fluid [kg/m^3^]	1.20
Reference Pressure [Pa]	10,1325
Acoustic Power [dB]	34.21
Shear Stress [Pa]	0 to 0.8

**Table 2:** Properties of parts manufactured using ABS and PLA polymers.

	ABS	PLA
Printing Temperature [°C]	210–250	205–225
Density (g/cm^3^)	1.04	1.24
Distortion Temperature [°C, 0.45 MPa]	98	52
Tensile Strength [MPa]	40	60
Elongation at break [%]	40	29
Impact Resistance [kJ/m^2^]	7.7	7.0
Tensile Elasticity Module [GPa]	0.65	1.08

**Table 3:** Prototyping parameters of different parts.

	Layer Height [mm]	Wall Line Count	Infill Density [%]	Printing Temperature [°C]	Build Plate Temperature [°C]	Support Structure	Thin Walls
Outside Air Multiplier Cilinder	0.12	8	100	230	70	Tree	Yes
Base Part	0.12	3	15	230	70	Tree	No
Motor Cage	0.12	8	15	230	70	Tree	No

## Data Availability

The data presented in this study are available on request from the corresponding author.
